# Antibody Kinetics after Three Doses of SARS-CoV-2 mRNA Vaccination in Patients with Inflammatory Bowel Disease

**DOI:** 10.3390/medicina59081487

**Published:** 2023-08-18

**Authors:** Evangelos Tsipotis, Ankith Maremanda, Laura Bowles Zeiser, Caoilfhionn Connolly, Sowmya Sharma, Sharon Dudley-Brown, Sarah Frey, Mark Lazarev, Joanna M. Melia, Alyssa M. Parian, Dorry L. Segev, Brindusa Truta, Huimin Yu, William A. Werbel, Florin M. Selaru

**Affiliations:** 1 Digestive Health Center, Augusta University, Augusta, GA 30912, USA; v.tsipotis@yahoo.com; 2 Hopkins IBD Center, Division of Gastroenterology, Department of Medicine, Johns Hopkins University School of Medicine, Baltimore, MD 21224, USA; amarema1@jhu.edu (A.M.); ssharm85@jh.edu (S.S.); sdudley2@jhmi.edu (S.D.-B.); mlazare1@jhmi.edu (M.L.); joanna.peloquin@jhmi.edu (J.M.M.); aparian1@jhmi.edu (A.M.P.); btruta1@jhmi.edu (B.T.); hyu20@jhmi.edu (H.Y.); 3 Department of Surgery, NYU Grossman School of Medicine, New York, NY 10016, USA; laura.zeiser@nyulangone.org; 4 Division of Rheumatology, Department of Medicine, Johns Hopkins University School of Medicine, Baltimore, MD 21224, USA; cconno15@jhmi.edu; 5 Department of Surgery, Johns Hopkins University School of Medicine, Baltimore, MD 21224, USA; sfrey6@jhmi.edu; 6 Department of Surgery Center for Surgical and Transplant Applied Research, NYU Langone Health, New York, NY 10016, USA; dorry@jhmi.edu; 7 Division of Infectious Diseases, Department of Medicine, Johns Hopkins University School of Medicine, Baltimore, MD 21224, USA; 8 Sidney Kimmel Comprehensive Cancer Center, Johns Hopkins University, Baltimore, MD 21224, USA

**Keywords:** COVID-19, SARS-CoV-2, vaccination, IBD

## Abstract

*Background:* The emergence of new SARS-CoV-2 variants calls for more data on SARS-CoV-2 mRNA vaccine response. *Aims:* We aimed to assess the response to a third mRNA vaccine dose against SARS-CoV-2 in inflammatory bowel disease (IBD) patients. *Methods:* This was a single-center, observational prospective study of IBD patients who received a third mRNA vaccine dose against SARS-CoV-2. Antibody titers were taken post-third-dose at one and three months using the Roche Elecsys anti-SARS-CoV-2-S enzyme immunoassay. Titers less than 0.8 units/mL were considered negative according to the manufactures. Titers between 0.8 units/mL and 250 units/mL were considered non-neutralizing. Titers greater than 250 units/mL were considered neutralizing. *Results:* Eighty-three patients were included, all of whom had detectable antibodies at 3 months post-third dose. A total of 89% showed neutralizing and 11% non-neutralizing titers. Participants with non-neutralizing titers were more likely to be on systemic corticosteroids (*p* = 0.04). Two participants seroconverted from negative to positive, whereas 86% with non-neutralizing titers boosted to neutralizing levels. Only one participant with neutralizing titers after a third dose had a decrease to a non-neutralizing level within 3 months. *Conclusions:* Our findings support the ongoing recommendations for additional doses in immunocompromised individuals. However, longitudinal studies with a greater-sized patient population are needed.

## 1. Introduction

Initial findings have demonstrated that the majority of patients with inflammatory bowel disease (IBD), including those with Ulcerative Colitis (UC) and/or Crohn’s Disease (CD), develop antibodies that persist for at least 6 months following a two-dose series of SARS-CoV-2 mRNA vaccines [1,2,3]. However, an attenuated antibody response (a lower concentration of anti-SARS-CoV-2 antibodies) has been observed in individuals who are taking corticosteroids, Tumor Necrosis Factor (TNF-ɑ) inhibitors, or combination therapies (the concurrent use of biologic medications and immunosuppressive treatments, which may include thiopurines like 6-mercaptopurine and azathioprine, as well as corticosteroids) [4,5,6]. These therapies are important for the management of IBD, and oftentimes, the magnitude of disease activity necessitates the continuation of these therapies regardless of vaccine prophylaxis of SARS-CoV-2. Consequently, the diminished antibody response following vaccination in these patients is concerning.

Moreover, subsequent to those studies, the emergence of the SARS-CoV-2 Omicron variant (BA.1/B.1.1.529) and its sublineages, which exhibit significant immune evasion and transmissibility, has been observed. The Omicron variant, identified in Africa in November 2021, possesses up to 32 mutations in the receptor-binding domain (RBD) and N-terminal domain of the spike protein, in comparison to the original variant. As the spike protein is the target of neutralizing antibodies, the presence of these mutations may enhance the virus’s ability to evade antibodies, thereby contributing to an increased viral fitness [7]. Recent studies have indicated that achieving neutralization in immunocompromised patients might require higher antibody titers [8,9].

In an effort to address waning antibody responses and enhance protection against novel SARS-CoV-2 variants such as Omicron and Delta (B.1.617.2) pseudo viruses, among others, the Advisory Committee on Immunization Practices (ACIP) of the Centers for Disease Control and Prevention (CDC) has recommended a third primary mRNA vaccine dose for individuals with moderate-to-severe immunocompromization. Additionally, booster doses are recommended for all individuals aged 5 years and above, at least four months after completing the initial mRNA vaccine series [10].

Initial studies in IBD patients show that the third dose may be safe to administer. A single-center study by Pellegrino et al. showed no repercussions in IBD patients to whom the BNT162b2 vaccine (Pfizer-BioNTech) was administered [11]. Additionally, Li et al.’s study showed that symptoms for IBD patients after a third mRNA vaccine dose tended to be milder than symptoms manifested after the second dose [12].

We conducted a single-center, observational prospective study to investigate 1-month and 3-month outcomes in IBD patients who received their third mRNA vaccine doses.

## 2. Materials and Methods

### 2.1. Patient Recruitment

Our study included English-speaking adult patients, defined as 18 years and older, who are living in the United States and have been diagnosed with IBD. These patients are maintained on immunosuppression treatment for IBD and/or have other coexisting conditions with an immune-mediated etiology. Additionally, they received three doses of FDA-approved mRNA vaccines (mRNA-1273 manufactured by Moderna or BNT162b2 manufactured by Pfizer-BioNTech). Those who had a prior COVID-19 infection were eligible to participate in the study. To track baseline demographics (age, sex, race) and clinical notes, including a list of medications patients were taking at the time of the study (including both IBD and non-IBD meds), we utilized a secure online form on a website maintained by the study team. Patients who received the third vaccine dose but had no prior information about their titer levels pre-third dose were not eligible for the study. Additionally, patients who did not speak English were excluded due to the lack of IRB approval for non-English consenting scripts. This study was approved by the Johns Hopkins Institutional Review Board (IRB00248540). Participants were notified about the study through alerts on their health portals (EPIC Systems Corporation) and by their physicians. They were informed that the study does not provide the vaccine, and study team members will not offer any guidance regarding whether one should receive the booster. Confirmation of the receipt of the third dose was obtained through communication with the patient and chart review. Informed consent was explicitly obtained from all participants before their inclusion in the study.

### 2.2. Titer Measurements

Antibody titers were measured using the Roche Elecsys anti-SARS-CoV-2-S enzyme immunoassay (EIA), which is a pan-immunoglobulin assay targeting the spike protein RBD. Titer measurements were performed at local clinical laboratories in Baltimore, Maryland, USA (Laboratory Corporation of America Holdings-LabCorp, Burlington, NC, USA). Measurements were taken before administration of the third mRNA vaccine dose, as well as one and three months after administration of the third dose. Titers less than 0.8 units/mL were considered negative according to manufacturer guidelines, and the assay’s saturation limit was 2500 units/mL. Based on live virus-neutralizing antibody data in immunosuppressed populations, titers ranging from 0.8 to 250 units/mL were considered non-neutralizing, while titers greater than 250 units/mL were considered neutralizing [7,8].

### 2.3. Software Used

Data analysis was conducted using STATA software version 17.0 (StataCorp, College Station, TX, USA). Study data, including baseline demographics and medication records, were collected and managed using REDCap secure electronic data capture tools (version 11.0.3) hosted at Johns Hopkins University (Baltimore, MD, USA). Tables were generated using Microsoft Word.

## 3. Results

In our study, 83 IBD patients completed a three-dose SARS-CoV-2 mRNA vaccination regimen and had paired antibody testing performed prior to the administration of the third dose and at one and three months following the third vaccination dose (Table 1 and Table 2). The median age of participants was 45.2 years, with a female predominance (72.2%). A total of 28.9% of patients in the study had an additional co-existing diagnosis of rheumatic disease (RMD).

The majority of patients (57.8%) received mRNA1273 (Moderna, Cambridge, MA, USA) as their third dose, with a mean (SD) interval of 154 (46.9) days from the second dose. The remaining 42.2% received BNT162b2 (Pfizer-BioNTech, Mainz, Germany) with a mean (SD) interval of 172 (39.8) days after the second dose. These findings indicate that most patients did not receive the third dose within one month after the administration of the second dose, as recommended by the CDC for individuals with moderate-to-severe immunocompromization (See Table 1 for details).

Among those who had non-neutralizing antibody levels, the majority were vaccinated with Moderna (five participants, 55.6%) and the mean (SD) time from second to third dose was 154 (46.9) days (Table 1). In this antibody titer group, five (55.6%) were being treated with anti-TNF-a inhibitors and one participant (11.1%) was on combination therapy of a systemic steroid with anti-TNF-a. Two participants (22.2%) were on mycophenolate mofetil.

Amongst the 83 participants, 1 participant (11.1%) with low titers had a flare post-vaccination with a third dose. No other adverse events were reported amongst our participants.

All participants with titers available at 3 months post-third dose had antibody titers checked before receiving the third dose. Table 2 summarizes the kinetics of antibody titers before and after the third dose. Prior to a third dose, 82 (98.8%) participants had detectable antibodies, including 42 (50.6%) with non-neutralizing titers, and only 2 (2.4%) had negative titers before the third dose. One patient was on infliximab monotherapy, and the other patient was on steroids, mycophenolate mofetil, and tacrolimus. Both patients developed positive but non-neutralizing antibody levels after the third dose.

At both 1 and 3 months post-third dose, 100% of participants had detectable antibodies, of whom 74 (89.2%) showed neutralizing titers and 9 (10.8%) showed non-neutralizing titers at 3 months (Table 2). Notably, the two participants who seroconverted from negative to positive after a third vaccine dose demonstrated non-neutralizing titers at 3 months, whereas 36 out of 42 participants (86%) with non-neutralizing titers boosted to neutralizing levels. Only 1 out of 40 participants (2.5%) attaining neutralizing titers after a third vaccine dose demonstrated a wane to non-neutralizing level by 3 months.

In contrasting antibody response groups, baseline characteristics were similar, as was the use of TNFa inhibitor therapy. Participants with non-neutralizing titers, however, were more likely to be taking systemic corticosteroid therapy (5/9 vs. 8/74, *p* = 0.04) and have peri-vaccination immunosuppressive held (4/9 vs. 10/74, *p* = 0.038). IBD flares were reported in one (11.1%) of the non-neutralizing titer group and three (4%) of the neutralizing titer group.

## 4. Discussion

In this study, we observed a neutralizing anti-RBD antibody titer response in the majority of IBD patients (89.2%) who received a third dose of an FDA-approved SARS-CoV-2 mRNA vaccine at least 3 months post-vaccination, using the currently recommended definition of high antibody titers. None of the participants had negative titers after receiving the third dose. Two participants had negative titers before the third dose but developed antibodies after receiving the vaccine. Only one participant (2.5%) with neutralizing titers before the third dose experienced a decrease to non-neutralizing titers at 3 months after vaccination. Two patients (2.4%) had non-neutralizing titers prior to the booster dose, and their titers remained non-neutralizing even after vaccination. These patients were either on infliximab or a combination of mycophenolate mofetil and tacrolimus. Among the four participants (4.8%) who experienced a flare, three had neutralizing titers. It is currently unclear why the majority of patients who flared were in the neutralizing titer group. One possible explanation could be that, compared to the low titer group, a lower proportion of patients in the high titer group were on anti-TNF-a therapy or systemic corticosteroids, indicating a possible poor control of their disease. Unfortunately, we did not have any data regarding the disease severity and activity among the groups. Interestingly, participants with non-neutralizing titers were more likely to be taking systemic corticosteroid therapy. Although there is limited existing literature on this topic, one systematic review showed that risk factors for not responding to SARS-CoV-2 vaccination included systemic corticosteroid usage [13].

Other studies have reported similar outcomes to ours. A multicenter prospective study involving the University of Wisconsin-Madison (Madison, WI, USA) and Mayo Clinic (Jacksonville, FL, USA) demonstrated that all IBD patients who received a third SARS-CoV-2 mRNA vaccine dose exhibited a humoral immune response [14]. Additionally, another study showed that IBD patients on similar treatment regimens to those in our study were able to mount a serological response comparable to a control group following the administration of a third dose [15]. A study focusing on patients with autoinflammatory rheumatic and musculoskeletal diseases also showed an increased humoral response in the majority of participants following the administration of a third SARS-CoV-2 mRNA vaccine dose [16].

Preventative health screening, including updated vaccinations for vaccine-preventable diseases, is a crucial aspect of IBD patient management. Providers who care for the IBD community must address the concerns and hesitations surrounding the SARS-CoV-2 vaccine. One study aimed at assessing the sentiments of IBD patients regarding the vaccine found that 33% of those surveyed were hesitant to get vaccinated primarily due to concerns about vaccine safety and efficacy [17]. We demonstrated that the administration of a third mRNA vaccine dose resulted in a neutralizing response in the majority of IBD patients, confirming the efficacy of the vaccine. Our results align with numerous other studies examining IBD patients receiving a third SARS-CoV-2 mRNA vaccine, providing consistent evidence of a humoral immune response. We hope that this can contribute to increasing confidence in SARS-CoV-2 vaccination for populations with IBD.

Some limitations of our study include a small sample size and a lack of racial and ethnic diversity within our patient population, with the majority of participants being white.

Furthermore, this study is limited by the absence of formal neutralization testing against variants of concern and the lack of anti-nucleocapsid testing to monitor subclinical infections or prior exposure. Additionally, the exact vaccine doses administered were not ascertainable. No testing was conducted to exclude breakthrough infections that could have resulted in sustained antibody titers in some participants. Longitudinal studies that include a diverse representation of IBD patients across the spectrum of disease activity are necessary to comprehensively assess the depth and durability of immune responses in this complex group with varying phenotypes.

Ultimately, our findings are consistent with other studies that have examined anti-RBD kinetics after both two and three doses of mRNA vaccination in IBD patients, as well as patients with other autoinflammatory diseases. These studies demonstrate that mRNA vaccines are safe for use in the IBD population [5,14,15,16].

## 5. Conclusions

In conclusion, the results of our study support the ongoing recommendation of the CDC for additional doses of SARS-CoV-2 mRNA vaccines in immunocompromised patients. While these initial results are encouraging and provide insight into the responsiveness of patients with IBD to a third mRNA vaccine dose, more comprehensive research involving a larger patient population and a longer time span is needed.

## Figures and Tables

**Table 1:** Demographic and clinical characteristics of IBD patients after the administration of three-dose SARS-CoV-2 mRNA vaccination regimen.

	Non-Neutralizing Antibody Levels (*n* = 9) ^1^	Neutralizing Antibody Levels (*n* = 74) ^1^
Age, mean (SD)	50.3 (13.2)	47.2 (13.4)
Age, median (MAD) Female sex, no. (%)	44.8 (10.0) 5 (55.6)	45.2 (10.2) 55 (74.3)
Non-white, no. (%)	0	4 (5.4)
RMD diagnosis, no. (%) ^2^		
Lupus	0	0
Arthritis	2 (22.2)	14 (18.9)
Sjogrens	1 (11.1)	1 (1.4)
Myositis	2 (22.2)	1 (1.4)
Scleroderma	1 (11.1)	0
Vasculitis	1 (11.1)	0
Other	0	1 (1.4)
Total	7 (77.8)	17 (23.0)
Non-Biologics		
Meds used in IBD, no. (%)		
5-ASA ^3^	0	2 (2.7)
Thiopurine ^4^	0	14 (18.9)
Methotrexate	1 (11.1)	1 (1.4)
Tofacitinib	0	2 (2.7)
Non-IBD meds, no. (%)		
Hydroxychloroquine ^5^	0	3 (4.0)
Mycophenolate ^6^	2 (22.2)	2 (2.7)
Tacrolimus	1 (11.1)	1 (1.4)
Cyclosporine	0	1 (1.4)
Leflunomide	0	1 (1.4)
Biologics		
TNF inhibitor ^7^	5 (55.6)	37 (50.0)
TNF inhibitor monotherapy	4 (44.4)	26 (35.1)
Ustekinumab	0	16 (21.6)
Vedolizumab	0	1 (1.4)
Etanercept	0	1 (1.4)
Corticosteroids		
Budesonide	0	4 (5.4)
Systemic Corticosteroid ^8^	5 (55.6)	8 (10.8) *
Combination therapy		
TNF inhibitor ^7^ and thiopurine ^4^ or methotrexate	0	7 (9.4)
TNF inhibitor ^7^ and corticosteroid ^8^	1 (11.1)	2 (2.7)
Medication held before vaccine, no. (%)		
Yes	4 (44.4)	10 (13.5) *
Vaccine, no (%)		
BNT162b2	4 (44.4)	31 (41.9)
mRNA-1273	5 (55.6)	43 (58.1)
Time from second to third dose (SD)	146 (30.5)	172 (40.1)
Mean (SD) anti-RBD	115.2 (68.8)	1980.4 (756.9)
Flare, no. (%)	1 (11.1)	3 (4.0)

SD (Standard Deviation), MAD (Median Absolute Deviation), RMD (Rheumatic and Musculoskeletal Diseases), 5-ASA (5-aminosalicylic acid), TNF (Tumor Necrosis Factor). ^1^ Percentages in the columns are represented as percent of each category in the overall column. Negative antibody response was defined as Roche Elecsys anti-RBD pan Ig less than 0.8 u/mL. Non-neutralizing antibody titers were defined as anti-RBD pan Ig 0.8 to 250 u/mL. Neutralizing antibody titers defined as anti-RBD pan Ig greater than or equal to 250 u/mL. No participant had negative antibody titers at 3 months post-vaccination with a third dose. ^2^ Participants also have a diagnosis of systemic lupus erythematosus, myositis, Sjὅgren’s syndrome, rheumatoid arthritis, systemic sclerosis, ankylosing spondylitis, reactive arthritis, inflammatory bowel disease associated arthritis, psoriatic arthritis, polyarteritis nodosa, polymyalgia rheumatica, temporal arteritis, Behcet’s syndrome, eosinophilic granulomatosis polyangiitis, Henoch–Schonlein purpura, granulomatous polyangiitis, Takayasu’s arteritis, or microscopic polyangiitis. ^3^ 5-ASA includes prescription of mesalamine, sulfasalazine, and olsalazine. ^4^ Thiopurines include prescription of azathioprine and 6-mercaptopurine (6-MP). ^5^ Hydroxychloroquine includes prescription of hydroxychloroquine and chloroquine. ^6^ Mycophenolate includes prescription of mycophenolate mofetil and mycophenolic acid. ^7^ TNF-a inhibitors include prescription of adalimumab, infliximab, certolizumab-pegol, golimumab, and etanercept. ^8^ Other corticosteroid includes prescription of prednisone and prednisone equivalents (such as prednisolone). * Indicates that the two groups were statistically significantly different using Fisher exact test. *p*-value for systemic cosrticosteroids was 0.004 and for holding medication was 0.038.

**Table 2:** Antibody response against SARS-CoV-2 spike protein three months following two doses of SARS-CoV-2 vaccine in patients with IBD, stratified by a one-month antibody response after mRNA three-dose series.

		Antibodies Three Months after D3			
Antibodies-before D3		Negative	Non-neutralizing titers	Neutralizing titers	Total
	Negative	0	2	0	2
	Non-neutralizing titers	0	6	36	42
	Neutralizing titers	0	1	38	39
	Total	0	9	74	83

Negative response was defined as Roche Elecsys anti-RBD pan Ig less than 0.8 u/mL. Non-neutralizing antibody titers were defined as anti-RBD pan Ig 0.8 to 250 u/mL. Neutralizing antibody titers were defined as anti-RBD pan Ig greater than or equal to 250 u/mL. A total of 74 participants (89.2%) exhibited neutralizing antibody titers, while 9 participants (10.8%) showed positive but non-neutralizing antibody titers at three months following administration of a third vaccine dose. Among those with neutralizing antibody levels, the most commonly prescribed medication was TNF-a inhibitors, which were being used by 37 patients (50.0%). Treatment with thiopurines was observed in only 14 patients (18.9%), corticosteroids in 8 patients (10.8%), and budesonide in 4 patients (5.4%). Additionally, 7 participants (9.5%) with neutralizing antibody levels were receiving a combination of anti-TNF-a therapy with thiopurines or methotrexate, and 2 participants (2.7%) were on a combination of an anti-TNF-a medication with systemic corticosteroids. Three patients (3.6%) with high antibody titers experienced a flare-up following vaccination.

## Data Availability

The data underlying this article will be shared on reasonable request to the corresponding author.
